# Presence of the dissociative subtype of PTSD does not moderate the outcome of intensive trauma-focused treatment for PTSD

**DOI:** 10.1080/20008198.2018.1468707

**Published:** 2018-05-18

**Authors:** Harmen A. Zoet, Anouk Wagenmans, Agnes van Minnen, Ad de Jongh

**Affiliations:** a Research Department, PSYTREC, Bilthoven, The Netherlands; b Behavioural Science Institute (BSI), Radboud University Nijmegen, Nijmegen, The Netherlands; c Academic Centre for Dentistry Amsterdam (ACTA), University of Amsterdam and VU University Amsterdam, Amsterdam, The Netherlands; d School of Health Sciences, University of Salford, Manchester, UK; e Institute of Health and Society, University of Worcester, Worcester, UK

**Keywords:** Posttraumatic stress disorder, dissociative subtype of PTSD, intensive trauma-focused treatment, prolonged exposure, EMDR therapy, stabilization phase, dissociation, trastorno de estrés postraumático, subtipo disociativo de TEPT, tratamiento intensivo centrado en el trauma, exposición prolongada, Terapia EMDR, fase de estabilización, disociación, 创伤后应激障碍, PTSD的分离亚型, 以创伤为中心的强化治疗, 延长暴露, EMDR疗法, 稳定阶段, 解离, • Patients with the dissociative subtype of PTSD (DS) differed from those not having DS in the severity of PTSD levels., • DS and non-DS patients showed similar levels of improvement after trauma-focused therapy., • Intensive treatment programmes can be quite effective even for patients with high levels of dissociation.

## Abstract

**Background**: There is a widely-held belief in the trauma field that the presence of dissociative symptoms is associated with poor treatment response. However, previous research on the effect of dissociation in treatment outcomes pertained to specific patients and trauma populations.

**Objective**: To test the hypothesis that the presence of the dissociative subtype of PTSD (DS) would have a detrimental effect on the outcome of an intensive trauma-focused treatment programme.

**Methods**: PTSD symptom scores (Clinician Administered PTSD Scale [CAPS] and PTSD Symptom Scale Self-Report [PSS-SR]) were analysed using the data of 168 consecutive patients (70.6% female) who had been exposed to a wide variety of multiple traumas, including childhood sexual abuse, and of whom 98.2% were diagnosed with severe PTSD (CAPS > 65). Most of them suffered from multiple comorbidities and 38 (22.6%) met the criteria for DS. They took part in an intensive trauma-focused treatment programme for PTSD. Pre- and post-treatment differences were compared between patients with and without DS.

**Results**: Large effect sizes were achieved for PTSD symptom reduction on CAPS and the PSS-SR, both for patients with DS and those without. Although patients with DS showed a significantly greater PTSD symptom severity at the beginning, and throughout, treatment, both groups showed equal reductions in PTSD symptoms. Of those who met the criteria for DS, 26 (68.4%) no longer fulfilled the criteria for this classification after treatment.

**Conclusion**: The results provide no support for the notion that the presence of DS negatively impacts trauma-focused treatment outcomes. Accordingly, PTSD patients with DS should not be denied effective trauma-focused treatments.

## Abstract (es)

**Introducción:** Existe una creencia generalizada en el campo del trauma de que la presencia de síntomas disociativos se asocia con una peor respuesta al tratamiento. Sin embargo, las investigaciones previas sobre el efecto de la disociación en los resultados del tratamiento se han realizado con pacientes y poblaciones de trauma específicos.

**Objetivo:** probar la hipótesis de que la presencia del subtipo disociativo de TEPT (SD) tendría un efecto perjudicial sobre el resultado de un programa de tratamiento intensivo centrado en el trauma.

**Métodos:** se analizaron las puntuaciones de síntomas de TEPT (CAPS y PSS-SR) utilizando los datos de 168 pacientes consecutivos (70,6% mujeres), que habían estado expuestos a una amplia variedad de traumas múltiples, incluido el abuso sexual infantil, y de los cuales 98,2% fueron diagnosticado con TEPT severo (CAPS > 65). La mayoría sufría de múltiples comorbilidades y 38 (22,6%) cumplían los criterios para el SD. Estos pacientes participaron en un programa de tratamiento intensivo centrado en el trauma para el TEPT. Las diferencias pre y post tratamiento se compararon entre los pacientes con y sin SD.

**Resultados:** Se obtuvieron considerables tamaños del efecto para la reducción de los síntomas de TEPT en el CAPS y el PSS-SR, tanto para los pacientes que experimentan SD como los que no. Aunque los pacientes con el SD mostraron una gravedad de síntomas de TEPT significativamente mayor al inicio, y durante todo el tratamiento, ambos grupos mostraron reducciones semejantes en los síntomas de TEPT. De los participantes que cumplían criterios para el SD, el 26 (68.4%) ya no cumplían dichos criterios para esta clasificación después del tratamiento.

**Conclusión:** Los resultados no respaldan la idea de que la presencia del SD tenga un impacto negativo en los resultados del tratamiento centrado en el trauma. En consecuencia, no se les debe negar a los pacientes con TEPT con SD tratamientos efectivos centrados en el trauma.

## Abstract (zh)

背景：创伤领域普遍认为，分离症状的存在与治疗反应不佳有关。 然而，以前关于分离治疗结果影响的研究涉及的是特定的患者和创伤人群。

目的：检验PTSD（DS）分离亚型会对以创伤为中心的强化治疗计划的结果产生不利影响的假设。

方法：使用168名持续患者（70.6％女性）的数据分析PTSD症状评分（CAPS和PSS-SR）。这些患者曾暴露于多种创伤包括儿童期性虐待，其中98.2％被诊断为严重PTSD（CAPS> 65）。他们中的大多数患有多种合并症，38名（22.6％）符合DS的标准。 他们参加了针对PTSD的强化创伤治疗计划。 对治疗前和治疗后不同DS患者之间的差异进行比较。

结果：对于DS患者和无DS患者，CAPS和PSS-SR 显示PTSD症状呈现效应量较大的减轻。 虽然DS患者在开始和整个治疗过程中表现出显着更严重的PTSD症状，但两组均表现出PTSD症状相同程度的减少。 在达到DS标准的人群中，有26人（68.4％）在治疗后不再符合该分类标准。

结论：结果不支持DS的存在对创伤治疗结果产生负面影响的观点。 因此，具有DS的PTSD患者不应该被拒绝接受有效的以创伤为中心的治疗。

## 1. Introduction

Posttraumatic stress disorder (PTSD) is a highly burdensome mental disease, severely impacting the life of patients and their surroundings, as well as society in general (Calhoun, Beckham, & Bosworth, 2002; Kessler, 2000). According to international guidelines, trauma-focused psychotherapies (TFPs) are the first-choice treatments for PTSD (National Institute for Health and Clinical Excellence, 2005; World Health Organization, 2013). Recent meta-analyses show that TFPs are more likely to be effective in the treatment of PTSD than non-TFPs and psychopharmacological treatments, as the core of PTSD symptomatology is being targeted rather than merely blunting the expression of PTSD symptoms (Cusack et al., 2016; Lee et al., 2016). Of all TFPs, exposure-based cognitive behavioural therapies (e.g. prolonged exposure; Foa, Hembree, & Rothbaum, 2007) and eye movement desensitization and reprocessing (EMDR) therapy (Shapiro, 2001) have the strongest evidence base (Watts et al., 2013; World Health Organization, 2013).

Yet, when it comes to effectiveness, there is room for improvement. For example, a meta-analysis by Bradley, Greene, Russ, Dutra, and Westen (2005) indicated that 33% of the patients still meet the criteria of PTSD after completing some form of trauma-focused treatment. Another study found that up to 15% of those who developed PTSD were not likely to recover following treatment (Fletcher, Creamer, & Forbes, 2010). Although it is difficult to compare these numbers due to the varying definitions of improvement and recovery that were used, the message remains clear in that a substantial group of patients does not benefit from a TFP or still suffers from residual symptoms after treatment (Bradley et al., 2005).

One of the factors that is indicated as being negatively associated with reduced treatment results is dissociation. It has been argued that dissociation could hinder fear activation, which is considered to be a key mechanism of successful PTSD treatment (Cooper, Clifton, & Feeny, 2017; Ebner-Priemer et al., 2009; Littel, Remijn, Tinga, Engelhard, & van den Hout, 2017). In line with this reasoning, experts in the field of dissociation state that TFPs can lead to an ‘increase in PTSD and related symptoms, including dissociation, emotion dysregulation, and an increase in the patient’s overall distress and functional impairment’ (Lanius et al., 2010, p. 644). Indeed, a study among 137 individuals who had just recently been exposed to a traumatic event showed that dissociation prior to the first session of an early intervention (consisting of three 60-min sessions across three weeks) was associated with a reduced treatment response (Price, Kearns, Houry, & Rothbaum, 2014). Likewise, in a sample of 244 veterans who had received a standardized six-week residential treatment programme for PTSD consisting of individual trauma-focused CBT and group therapy sessions, Murphy and Busuttil (2015) found that higher levels of baseline dissociation were associated with worse post-treatment PTSD outcomes. Further, a study that evaluated data from 69 PTSD patients receiving EMDR therapy showed dissociation, as indexed by the Dissociation Experiences Scale, to be a significant predictor of non-response (Bae, Kim, & Park, 2016). However, in the latter study patients were given only four sessions of EMDR, which is commonly regarded as an inadequate dosage of active treatment. This might have negatively influenced the effects of patients with the dissociative subtype, who usually have more severe PTSD symptoms and are therefore in need of the full dosage of trauma-focused treatment. Other authors refer to opposing findings, suggesting that dissociative symptoms alleviate due to TFP treatment (Resick, Suvak, Johnides, Mitchell, & Iverson, 2012; van Minnen, Harned, Zoellner, & Mills, 2012), stating that patients with dissociative symptoms benefit similarly from TFP treatment compared to patients without these symptoms (Hagenaars, van Minnen, & Hoogduin, 2010).

In DSM-5, the ‘dissociative subtype of PTSD’ (DS) was included in the diagnostic criteria of PTSD diagnosis (American Psychiatric Association, 2013). This diagnostic category pertains to a subgroup of PTSD patients who exhibit key dissociative phenomena, i.e. depersonalization (the experience of feeling detached from one’s body) and/or derealization (the experience of unreality of surroundings). The decision to add DS to DSM-5 was partly based upon theoretical assumptions (Lanius, Brand, Vermetten, Frewen, & Spiegel, 2012; Wolf et al., 2012; Wolf, Miller, Harrington, & Reardon, 2012) and empirical evidence suggesting that individuals with DS show increased activation in the rostral anterior cingulate cortex and the medial prefrontal cortex (Lanius et al., 2010). This would lead to emotional overmodulation, thereby preventing emotional engagement with trauma-related material, making individuals with DS less likely to respond to TFPs. Accordingly, it is assumed that individuals experiencing depersonalization and/or derealization benefit most from skills training in affective and interpersonal regulation prior to TFP (Lanius et al., 2012). A study using a sample of 284 female active duty service members and veterans undergoing either prolonged exposure or present-centred therapy (Wolf, Lunney, & Schnurr, 2016) found that PTSD patients with DS showed poorer treatment response compared to PTSD patients who did not fulfil the criteria of DS. However, the magnitude of this effect was very small, which led the authors to conclude that exposure therapy is indeed effective in dissociative symptoms. These findings are consistent with the results of a secondary analysis of a treatment study of narrative exposure therapy (NET) and treatment as usual among severely traumatized asylum seekers and refugees, which showed that DS did not substantially moderate the treatment outcomes (Halvorsen, Stenmark, Neuner, & Nordahl, 2014). A recent study replicated these findings by generalizing the results to individuals with a severe psychiatric condition and performing a secondary analysis of a randomized clinical trial comparing prolonged exposure with EMDR therapy among PTSD patients with psychosis (van den Berg et al., 2015; van Minnen et al., 2016). Patients with and without DS showed a similar decrease in PTSD symptoms with large effect sizes observed in both groups. Because previous research on the effect of dissociation in treatment outcomes pertained to specific trauma populations (i.e. female veterans, refugees, psychotic patients), the results need further replication in a more heterogeneous sample in terms of patients and traumatic events.

The purpose of the present study was, therefore, to determine the impact of dissociation on PTSD treatment outcome using a sample of both women and men with PTSD as a result of a wide variety of traumatic events and suffering from multiple comorbidities. These patients underwent a short, highly intensive treatment programme consisting of two first-line TFPs for PTSD (prolonged exposure and EMDR therapy) which were applied without a preceding stabilization phase, and without explicitly addressing dissociation during treatment. Based upon the assumptions underlying the decision to include DS within DSM-5, it was hypothesized that patients who met the criteria for DS would respond significantly more poorly than those who did not. To our knowledge, previous studies did not examine whether the symptoms of DS are amendable to change. Therefore, an additional aim was to determine the degree of loss of DS diagnostic criteria following trauma-focused treatment.

## 2. Methods

### 2.1. Participants

Participants were patients who were enrolled in an intensive treatment programme for PTSD between August and December 2016 at the Psychotrauma Expertise Centre (PSYTREC) in the Netherlands. All of them were referred for treatment by their general practitioner, psychiatrist or another mental health care centre. The only service referral criterion was a (probable) diagnosis of PTSD. No preparatory interventions were offered beforehand. Less than 10% of the referred patients did not attend the first intake session, about half of them rescheduled the appointment though, indicating that the intensive treatment programme was acceptable for patients. A total of 182 patients entered treatment, of whom 177 (97.3%) gave informed consent for participation in clinical research. Three patients (1.7%) dropped out of treatment. These individuals were excluded from the analysis. For six participants, the post-treatment scores of the primary outcome measure, the Clinician Administered PTSD Scale (CAPS), were lacking. This resulted in a sample of 168 participants. Of the total sample, 146 (86.9%) had received prior psychological treatment; of them, 78 (46.4%) already received EMDR-therapy sessions and five (3.0%) prolonged exposure therapy prior to engaging in the intensive treatment programme. Table 1 provides an overview of the sample characteristics of the 168 participants.

**Table 1.** Sample characteristics (*N* = 168).

	DS	No DS
Mean age (*SD*)	36.05 (11.32)	38.78 (10.78)
Sex (% female)	81.6%	66.9%
PTSD severity (CAPS)		
Mild (score < 45)	0%	0%
Moderate (score 45–64)	2.6%	1.5%
Severe (score ≥ 65)	97.4%	98.5%
Trauma exposure		
Childhood sexual abuse	42.1%	43.8%
Adult sexual abuse	34.2%	28.5%
Physical abuse	73.7%	84.6%
Work-related trauma	26.3%	22.3%
Accidents, disasters or war violence	21.1%	27.7%
Comorbidity (MINI)		
Depressive episode	57.9%	72.3%
Dysthymia	55.3%	47.7%
Hypomania	2.6%	2.3%
Mania	5.3%	5.4%
Panic disorder	18.4%	17.7%
Agoraphobia	13.2%	23.1%
Social phobia	18.4%	36.2%
Obsessive compulsive disorder	7.9%	13.8%
Alcohol dependency	10.5%	17.7%
Suicidal risk (MINI)		
None	26.3%	19.2%
Low	15.8%	32.3%
Moderate	15.8%	18.5%
High	28.9%	27.7%

Percentages of *Comorbidity* and *Trauma exposure* do not add up to 100%, since individuals could have experienced multiple types of trauma and could suffer from multiple comorbidities. *Comorbidity* and *Suicidal risk* were assessed using the Mini-International Neuropsychiatric Interview (MINI), type of trauma was determined using a modified version of the Interview for Traumatic Events in Childhood (ITEC) and severity of PTSD symptoms was indexed using the Clinician Administered PSTD Scale (CAPS). Work-related trauma covers trauma exposure during military service, service in a police force, as a fire-fighter or whilst working in the mental health care.

### 2.2. Procedure

Pre-treatment assessment took place at the treatment centre, using the Dutch versions of CAPS (Blake et al., 1995; Hovens, Luinge, & van Minnen, 2005) and Mini International Neuropsychiatric Interview (MINI; Overbeek, Schruers, & Griez, 1999; Sheehan et al., 1998). Additionally, participants were asked to fill out the PTSD Symptom Scale Self-Report questionnaire (PSS-SR; Foa, Riggs, Dancu, & Rothbaum, 1993) and the modified Interview for Traumatic Events in Childhood (ITEC; Lobbestael, Arntz, Harkema-Schouten, & Bernstein, 2009). Inclusion criteria were: (1) being at least 18 years old, (2) having a diagnosis of PTSD according to the DSM-IV-TR (APA, 2000) as established with CAPS, (3) having sufficient knowledge of the Dutch language to be able to complete the assessments, (4) not being convicted for a sexual assault and (5) not having a history of a suicide attempt in the three months prior to treatment. If these criteria were met, the patient was invited to sign a treatment contract and informed consent for research purposes. The study was performed in accordance with the precepts and regulations for research as stated in the Declaration of Helsinki and the Dutch Medical Research on Humans Act (WMO) concerning scientific research. That is, all data were collected using the standard assessment instruments and routine outcome monitoring procedure of the PSYTREC mental health centre, the study lacked random allocation and no additional physical infringement of the physical and/or psychological integrity of the individual was to be expected.

After the intake sessions, the intensive eight-day treatment programme started. Nine days after treatment, patients returned to the centre for the post-treatment assessment, consisting of CAPS and PSS-SR. Assessors of both pre-treatment and post-treatment measures were blind to the study hypotheses.

### 2.3. Treatment

Treatment was provided in 2 × 4 consecutive days. Following the first four days of treatment patients went home for three days, and then returned to the clinic for the last four days of treatment. Each treatment day consisted of a morning session (i.e. 90 minutes of individual prolonged exposure therapy), an afternoon session (90 minutes of individual EMDR therapy) and an evening programme (two hours of psycho-education about PTSD), with six hours of sport activities in between.

In this study, prolonged exposure and EMDR therapy were combined. Given research suggesting that prolonged exposure and EMDR have different underlying working mechanisms (Lee, Taylor, & Drummond, 2006), we considered it likely that such a combination would increase the proportion of patients that responds to trauma-focused treatment and reduce the drop-out rate.

The purpose of psycho-education was to provide insight in PTSD symptoms, triggers and avoidance behaviours. No exercises regarding emotion regulation, relaxation or grounding were offered during these psycho-education sessions.

The sport activities were aimed at activation and were implemented because of its strong empirical support as an intervention for physical and mental health conditions in general, and for enhancement of trauma-focused treatment in PTSD specifically (Powers et al., 2015; Rosenbaum et al., 2015). Exercises within the sport programme varied from low to high intensity and were both indoor and outdoor (e.g. mountain biking, hiking, obstacle course, archery).

Therapy sessions were carried out by clinical psychologists who were trained in exposure therapy and EMDR therapy, and had received additional in-house training sessions specifically designed for this intensive treatment programme. Patients were treated by multiple, rotating therapists and their sessions and progress were discussed in daily meetings, which also contributed to assuring adherence to the treatment protocols.

EMDR therapy was provided according to guidelines by Shapiro (2001) and the Dutch version of the EMDR standard protocol (de Jongh & Ten Broeke, 2013), comprising an eight-phase psychotherapeutic approach. In EMDR therapy, patients envision the currently most distressing part of a traumatic event while burdening their working memory capacities by visually following finger movements of the therapist. To maximize the working memory load, therapists were allowed to add additional stimuli if needed, such as a light bar, clicking sounds and tactile stimulation via a pulsator. Cognitive interweaves, as described by Shapiro (2001), could also be employed when needed.

The prolonged exposure sessions followed a modified version of Foa’s prolonged exposure protocol (Foa et al., 2007). As outlined in Foa’s protocol, patients are exposed to the memories of traumatic events by means of imagining the traumatic event as vividly as possible and describing it aloud in the present tense and in detail. The processing of the traumatic memories is also included. Because of the intensive treatment format, no homework assignments were given. Sessions were therefore also not recorded. No in-vivo homework assignments were given. However, in vivo material, such as pictures, sound fragments, clothes and other objects that reminded the patient of the traumatic event, were incorporated into the prolonged exposure sessions.

### 2.4. Measurements

As a primary outcome measure, clinician-rated PTSD symptom severity was measured using the Dutch version of CAPS (Hovens et al., 2005), a well-validated semi-structured diagnostic interview (Hovens et al., 1994; Weathers, Keane, & Davidson, 2001). It comprises the 17 DSM-IV PTSD symptoms – clustered in three subscales (*Re-experiencing, Avoidance*, *Hyperarousal*) – measured over the past month using 5-point scales for frequency (ranging from 0 = ‘never’ to 4 = ‘almost daily’) and intensity (ranging from 0 = ‘none’ to 4 = ‘extremely’). Total severity scores were computed by adding up all frequency and intensity ratings across the 17 items (ranging from 0 to 136). Total severity scores of ≥ 65 indicated severe PTSD, scores between 45 and 65 indicated moderate symptoms of PTSD, and scores below 45 indicated mild or no symptoms of PTSD (Weathers et al., 2001). CAPS was administered at pre- and post-treatment. CAPS included two separate items representing the symptoms of the dissociative subtype: derealization and depersonalization. Using the conservative score rule of frequency ≥ 2 and severity ≥ 2 on at least one of these items, patients were classified as meeting or not meeting the criteria of the dissociative subtype (as per Nicholson et al., 2015).

Self-reported severity of PTSD symptoms was measured with PSS-SR (Arntz, 1993; Foa et al., 1993), a 17-item questionnaire that assesses the severity of PTSD symptoms in the past week based on the diagnostic criteria of the DSM–IV. PTSD symptom severity is rated on 4-point Likert scales from 0 (*not at all*) to 3 (*very much*) and scores per symptom add up to a total severity score ranging from 0 to 51, with higher scores suggesting higher PTSD severity. The internal consistency is considered satisfactory and the concurrent validity is considered good (Foa et al., 1993). PSS-SR was administered on the first day of treatment and at post-treatment.

The Dutch version of MINI (Overbeek et al., 1999; Sheehan et al., 1998) was used to establish comorbidity, i.e. additional DSM-IV axis-I disorders. MINI is a validated structured interview that has proven to be a reliable classification instrument (Lecrubier et al., 1997; Sheehan et al., 1998). Items are dichotomous (‘yes’ or ‘no’) and represent DSM-IV criteria. Individuals were diagnosed when they met sufficient criteria. MINI was also used to determine suicidal risk, which was categorized as low, moderate or high.

### 2.5. Statistical analyses

IBM SPSS Statistics for Windows (version 20) was used to perform a mixed-design ANOVA. The dissociative subtype of PTSD (DS) was used as dichotomous independent variable, and change in scores on both CAPS (pre- and post-treatment) and PSS-SR (at day 1 and post-treatment) as the dependent variable. Person mean imputation was performed when the percentage of missing data on the total number of items of a measure did not exceed 10% (Downey & King, 1998; Hawthorne & Elliott, 2005). If this was not the case, participants were excluded from analyses (Cohen, Cohen, West, & Aiken, 2003). For this reason, 10 participants were excluded in the analysis of the primary outcome measure CAPS, and 18 participants were excluded from PSS-SR analysis. To compare baseline differences in demographic variables (age, gender, type of trauma) between dissociative subtype conditions, chi square analyses were conducted. Cohen’s *d* effect sizes were interpreted according to Cohen’s (1988) rules of thumb. To assess changes in the presence of the dissociative subtype before and after treatment, a McNemar test was performed, a method suitable for comparison of paired nominal data. Paired samples *t*-tests were conducted to compare pre-treatment and post-treatment mean scores on the dissociation items. Preliminary analyses indicated no violations of the assumption of homogeneity of variance.

## 3. Results

### 3.1. Sample characteristics

Of all patients, 38 (22.6%) met DSM-5 criteria for DS. Of the nine patients who were not included in the analyses due to incomplete data or premature drop-out, eight did not meet the criteria of DS. For the other participant, pre-treatment data was missing. Patients with and without DS did not differ in age (*t*(166) = 1.36, *p* = .177) or gender (χ^2^(1) = 3.02, *p* = .082). Also, no differences were found regarding type of trauma exposure, i.e. sexual abuse (*χ*^2^ (1) = 1.33, *p* = .249), physical abuse (*χ*^2^ (1) = 2.75, *p* = .097), work related traumas (*χ*^2^ (4) = 4.03, *p *= .402), and accidents, disasters or war violence (*χ*^2^ (1) = 0.71, *p* = .399) were equally prevalent in both groups. Sample characteristics regarding comorbidities and PTSD severity can be found in Table 1.

### 3.2. Overall treatment outcome

A mixed-design ANOVA with CAPS total scores as dependent variable showed a significant, main effect of time, *F*(1, 166) = 259.11, *p *< .001, $n_{p}^{2}$ = .61, see also Table 2. Employing Schnurr and Lunney’s (2016) categories for clinical meaningful outcome, 84.1% of the participants showed response, 55.3% lost their PTSD diagnosis, and PTSD was in remission for 27.1% of the participants. A mixed-design ANOVA with the PSS-SR-scores as outcome variable also indicated a significant main effect of time, *F*(1, 156) = 164.61, *p* < .001, $n_{p}^{2}$ = .51, see Table 2.

**Table 2.** Mean pre-treatment and post-treatment CAPS scores and PSS-SR scores and outcome of mixed-design ANOVA.

Measure Subset	Pre-treatment score *M* (*SD*)	Post-treatment score *M* (*SD*)	*p*	ηp^2^
CAPS				
Total (*N* = 168)	93.66 (12.81)	44.23 (32.38)	< .001	.61
DS (*N* = 38)	97.31 (11.29)	54.97 (32.74)		
No DS (*N* = 130)	92.60 (13.07)	41.09 (31.71)		
PSS-SR				
Total (*N* = 158)	35.99 (6.73)	18.19 (13.32)	< .001	.51
DS (*N* = 36)	37.63 (6.77)	22.11 (13.11)		
No DS (*N* = 122)	35.49 (6.67)	17.03 (13.21)		

CAPS = Clinician Administered PTSD Scale, PSS-SR = PTSD Symptom Scale Self-Report; ηp^2^ = partial eta squared.

### 3.3. Impact of dissociative subtype on patients’ CAPS scores

**Figure 1.**
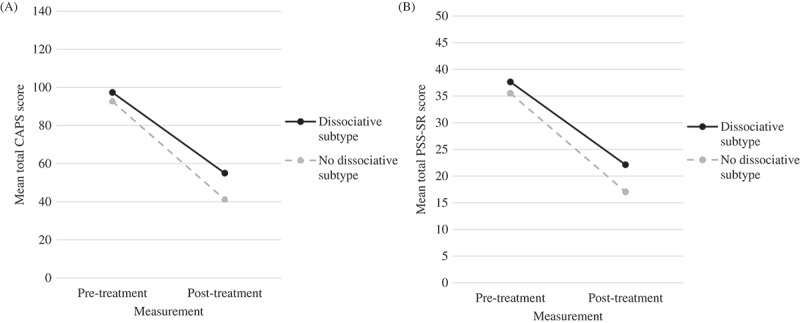
Mean total CAPS scores (A) and mean total PSS-SR scores (B) for patients with and without the dissociative subtype of PTSD.

The mixed-design ANOVA with CAPS total scores as dependent variable showed a significant main effect of group, *F*(1, 166) = 7.46, *p =* .007, $n_{p}^{2}$ = .04. However, the effect size was small; that is, the between-group differences in symptom severity were only for 4% associated with the presence or absence of DS. No interaction effect was found, *F*(1, 166) = 2.47, *p =* .118, $n_{p}^{2}$ = .02, which means that, albeit overall patients with DS scored significantly higher on both pre- and post-assessments than those without DS, patients with and without DS showed equal, significant reductions in CAPS scores over the course of treatment. Large effect sizes were achieved for PTSD symptom reduction on CAPS for patients both with DS (Cohen’s *d* = 1.73) and without (Cohen’s *d *= 2.12). The effect-size of the difference between the two groups was small (Cohen’s *d* = 0.43). Figure 1 illustrates the progression of mean total CAPS scores over time for both groups.

### 3.4. Impact of dissociative subtype on patients’ PSS-SR scores

The mixed-design ANOVA of PSS-SR scores showed a significant main effect of group, *F*(1, 156) = 5.96, *p* = .016, $n_{p}^{2}$ = .04. However, this effect was small; that is, the presence or absence of DS explained the between-group differences in symptom severity for only 4%. No interaction effect of time by group was found, *F*(1, 156) = 1.24, *p *= .268, $n_{p}^{2}$ = .01. Hence, despite the overall relatively higher pre- and post-treatment PSS-SR scores of those with DS, equal, significant reductions in PSS-SR scores were achieved throughout treatment for patients both with and without DS. Large effect sizes were achieved for PTSD symptom reduction on PSS-SR for patients both with DS (Cohen’s *d *= 1.49) and without (Cohen’s *d *= 1.76). The effect-size of the difference between the two groups regarding PSS-SR scores proved to be small (Cohen’s *d* = 0.38). Figure 1 illustrates the progression of mean total PSS-SR scores over time for both groups.

### 3.5. Change in the presence of the dissociative subtype

Of the 38 participants who met the criteria for DS, 26 (68.4%) no longer met these criteria following treatment. For those who initially met the criteria for DS, depersonalization was significantly less frequent (*t*(37) = 3.68, *p* = .001) and less intense (*t*(37) = 5.32, *p* < .001) after completing the treatment programme. Furthermore, derealization also significantly decreased in frequency (*t*(37) = 2.60, *p* = .013) and intensity (*t*(37) = 4.48, *p* < .001), see also Table 3.

**Table 3.** Changes in mean scores on the depersonalization and derealization items of CAPS for those who initially met the criteria for DS.

	Pre-treatment *M* (*SD*)	Post-treatment *M* (*SD*)	*P*	Cohen’s *d*
Depersonalization frequency	1.67 (1.35)	0.67 (1.22)	.001	0.78
Depersonalization intensity	1.75 (1.32)	0.50 (0.91)	< .001	1.10
Derealization frequency	1.72 (1.47)	0.89 (1.39)	.013	0.58
Derealization intensity	1.64 (1.40)	0.58 (0.91)	< .001	0.90

When the scoring rule of frequency ≥ 2 and intensity ≥ 2 was met on at least one of the dissociation items, participants were classified as meeting the criteria for the dissociative subtype.

## 4. Discussion

The present study examined whether the presence of DS has a detrimental effect on the outcome of a first-line trauma-focused treatment programme. Even though most patients in this study suffered from severe PTSD and multiple comorbidities, overall, a large and significant reduction in PTSD symptoms was achieved after treatment. More specifically, more than 80% showed a clinical meaningful response, whereas more than half of the patients lost their PTSD diagnosis. Over the course of treatment, the severity scores of both DS and non-DS groups decreased from severe PTSD to mild or no PTSD, as indexed by both clinician-administered and self-report measures. Most importantly, the decline in PTSD symptom severity was similar for individuals with DS and for those without DS. Hence, the results of the present study do not provide support for the notion that the presence of DS has a negative impact on the outcome of first-line, trauma-focused treatments for those suffering from severe PTSD.

The present findings are at odds with prevailing theoretical assumptions in the trauma field that individuals who experience a high level of state dissociation would do worse in trauma-focused treatments than other groups of patients (Lanius et al., 2010, 2012). It could be argued that physical activity is a useful type of ‘stabilizing work’, in that it might help patients to re-regulate if they become dissociated or overly emotional during or after trauma focused treatment. For instance, there is evidence indicating that sport has a positive effect on both symptoms of PTSD and depressive symptoms, and that physical activity might play a role in the multidisciplinary treatment of PTSD (Rosenbaum et al., 2015). Although further research about the specific role that physical activity plays in our treatment programme is needed, it should be noted that the results are consistent with a number of recent studies that did not incorporate any physical activity within their treatment. For example, those with women undergoing Cognitive Processing Therapy (CPT; Resick et al., 2012), female veterans and active duty service members undergoing Prolonged Exposure (PE; Wolf et al., 2016), individuals suffering from psychosis of schizophrenia who were treated with PE or EMDR Therapy (van Minnen et al., 2016) and asylum seekers and refugees who received NET (Halvorsen et al., 2014). The latter study demonstrated that 50% of the patients with severe derealization, and 50% showing severe depersonalization at pre-treatment, achieved clinically significant change in their CAPS total scores following treatment (Halvorsen et al., 2014). Interestingly, our results mimicked the findings of van Minnen et al. (2016) who also found that individuals with DS reported significantly greater PTSD symptom severity at the beginning, while this difference remained constant throughout treatment. To this end, it should be noted that, on average, the patients with DS in the current study still displayed a moderate level of PTSD symptoms after treatment. This suggests that the presence of dissociative symptoms demands a longer treatment duration or other treatment approaches to compensate for a higher initial PTSD severity, for example by specifically addressing patients’ fears of decompensation as a way to circumvent the occurrence of dissociative phenomena.

An intriguing finding of the present study is that, after treatment, a majority of patients with DS did not meet the criteria of this condition anymore, albeit dissociative symptoms were not explicitly addressed during treatment. This is in line with previous clinical trials, in which trauma-focused treatments were successful in reducing PTSD symptoms and dissociative symptoms concurrently (Hagenaars, van Minnen, Hoogduin, & Verbraak, 2009; Resick et al., 2012; van Minnen et al., 2016; Wolf et al., 2016). The large treatment-related change in the presence of DS, and that DS and non-DS patients showed similar levels of improvement after trauma-focused therapy, might have implications for both the reliability and the validity of this subtype of PTSD. This is particularly relevant in light of the suggestion that the aetiology of DS might be based upon distinct genetic vulnerabilities (Wolf et al., 2014), suggesting DS to be a trait rather than a state dependent subtype, as our data imply.

Strengths of the current study include the use of both clinician-administered and self-administered PTSD measures, and the diversity of the sample regarding gender, age and nature of the traumatic experiences. This study also faced several limitations. Firstly, insufficient follow-up data was available for analyses due to the relatively short existence of the treatment centre. Clearly, future studies should include follow-up data to be able to examine the course of the dissociative symptoms long after treatment because between-group differences might become apparent during follow-up (Cloitre et al., 2010). Secondly, the sample was dichotomized into either fulfilling DSM-5 criteria of a dissociative subtype or not, whereas no separate measures pertaining to dissociative features were included in the current study. Although derealization and depersonalization were indexed using the same items from CAPS as used in most previous research (Armour, Karstoft, & Richardson, 2014; Halvorsen et al., 2014; Wolf et al., 2012, 2016), the inclusion of a more elaborate measure of dissociative phenomena, such as the Multiscale Dissociation Inventory (Briere, 2002), would allow for a more in-depth analysis of gradations and alterations in dissociative symptoms. Thirdly, it could be considered a methodological weakness to not include an analysis on the follow-up data. However, the primary aim of the study was not to show that the intensive treatment programme was effective, but rather to examine whether DS moderated the treatment outcome at post-treatment. In some of the referenced studies in our introduction, follow-up results were included, and in none of these cases the effects differed from the post-treatment results, indicating that the results obtained at post-treatment are highly stable across time (e.g. Hagenaars et al., 2010; Resick et al., 2012). Also, studying only post-treatment outcomes offers the advantage of analysing moderating effects in a highly controlled treatment period, because we were able to constantly monitor and check that no other treatment elements than intended were added. In contrast, studying moderators on follow-up effects is less controlled, since patients may be undergoing additional interventions, including stabilizing treatments following the conclusion of the trauma-focused treatment. Fourth, our prolonged exposure protocol did not include homework assignments. Although our results are in line with findings from studies that used regular prolonged exposure including homework, we do not know how this may have influenced our positive results. Finally, the study lacked randomization, which prohibits meaningful conclusions regarding the superiority of either intensive, trauma-focused treatment or a phase-based treatment approach (see de Jongh et al., 2016).

In conclusion, besides evidence supporting the effectiveness of a short, intensive treatment programme consisting of a combination of two trauma-focused therapies without a prior stabilization phase, the present study results provide further support for the notion that the presence of DS does not moderate the outcome of trauma-focused treatments for PTSD. To this end, the results converge to suggest that excluding individuals with DS from trauma-focused treatment, or to include an initial stabilization phase to train emotion regulation skills prior to trauma-focused treatment, is generally not warranted (de Jongh et al., 2016; van Minnen, Arntz, & Keijsers, 2002; van Minnen et al., 2012).
